# Bela Julesz in Depth

**DOI:** 10.3390/vision3020018

**Published:** 2019-05-08

**Authors:** Thomas V Papathomas, Kazunori Morikawa, Nicholas Wade

**Affiliations:** 1 Laboratory of Vision Research, Center for Cognitive Science, and Department of Biomedical Engineering, Rutgers University, Piscataway, NJ 08854, USA; 2 School of Human Sciences, Osaka University, Suita, Osaka 565-0871 Japan; 3 Department of Psychology, University of Dundee, Nethergate, Dundee DD1 4HN, UK

**Keywords:** depth perception, stereopsis, texture, perceptual portrait, random-dot stereograms

## Abstract

A brief tribute to Bela Julesz (1928–2003) is made in words and images. In addition to a conventional stereophotographic portrait, his major contributions to vision research are commemorated by two ‘perceptual portraits’, which try to capture the spirit of his main accomplishments in stereopsis and the perception of texture.

Binocular vision has been a topic of enquiry for at least two millennia but linking retinal disparity to depth perception is much more recent. Almost two centuries ago, the invention of the stereoscope by Wheatstone [1] revolutionized the experimental investigation of binocular vision. Separating the perception of depth from object recognition (with random-dot stereograms [2]) heralded a second revolution. It is then fitting that a scientist who contributed so much to our understanding of stereoscopic vision should be presented stereoscopically, as in Figure 1. Most significantly, Bela Julesz is also represented in two ‘perceptual portraits’. Their aim is to combine his portrait with graphical elements that reflect the contributions that he made to the study of mind and behavior—after the manner of those displayed in Wade’s book [3]. As in that book, this brief article pays tribute to Bela Julesz—shortly after the fifteenth anniversary of his death—in words as well as images.

In Figure 1 and in the subsequent figures the stereoscopic portraits are presented in three ways. The upper image is an anaglyph combining the left and right images; the stereoscopic depth can be seen with the red filter of red/cyan glasses in front of the left eye and the cyan filter before the right eye. Beneath the anaglyphs are three images—two for the left eye (L) flank that for the right (R), forming the triplet LRL. Combining the central and right image by “crossed convergence” will yield conventional stereoscopic depth. Pseudoscopic viewing, i.e., swapping the two eyes’ images (see caption for Figure 1) does not elicit a percept of a concave mask, as the binocular disparity signals would indicate; instead, most observers perceive a normal convex face, i.e., they obtain the “hollow-mask illusion” [4,5].

Julesz is best known for his invention of the random-dot stereogram (RDS) and its use in numerous areas of vision research, as detailed in his classic monograph [2]. Binocular vision is one of the oldest topics of investigation in vision and Julesz provided a solution to a problem appreciated by Wheatstone [1,6]—how can stereoscopic vision be examined independently of monocular object recognition? Wheatstone used outline figures to overcome the difficulty but the problem was more acute when viewing objects with his pseudoscope; reversed disparity did not overrule object properties. The RDS dispelled the conflation of object recognition and stereoscopic vision thereby heralding a new paradigm in the study of depth perception. Julesz referred to this as *cyclopean* perception and his portrait is combined with random-dot patterns in Figure 2. His contributions to the understanding of stereopsis are universally accepted, and eventually led to powerful new methods for studying disparity-tuned neurons at the very early stages of the visual cortex [7], as he had predicted. In addition to depth, Julesz’s random-dot patterns were almost as influential in the study of visual motion. As Chubb [8] put it, “the random-dot cinematogram [9] revolutionized the way in which we now think about motion perception,” by providing Braddick [10] with the tools for his seminal studies on ‘short-range’ and ‘long-range’ motion. In addition, Julesz and his colleagues provided direct evidence for the role of color in motion [11].

Texture perception is another area in which Julesz’s work had a major impact. Since he had always held that texture is processed at the very early stages of the visual pathways, one of his favorite expressions was that texture is a “royal road to preattentive vision.” His pioneering article [12] was the first to attempt a systematic approach in this area. The formulation of his texton theory required quite sophisticated mathematics—see, for example, [13] (pp. 131–142)—and acted as a catalyst for significant progress in texture perception. In addition, Julesz and his colleagues made important contributions in visual attention, learning and plasticity, and perceptual organization in spatial vision (e.g., [14]). Finally, he was among the first vision researchers to use the concept of cooperative systems in perception [15], i.e., systems that, according to Julesz, “exhibit order-disorder transitions, multiple ordered states, and hysteresis” [13] (p. 196). The short length of this article allows space only for major contributions that had a lasting impact on vision research. The interested reader can find more information in Julesz’s second monograph [13], which is a scientific autobiography, and in [16], which contains the proceedings of a 1993 two-day conference that celebrated Julesz’s 65th birthday and attracted about 200 participants from around the world.

If we were forced to come up with a title for Julesz, in Wade’s style [3], the choice is easy. Julesz was a *reductionist* par excellence, as the following comments from his monograph indicate: “I regard as ‘scientific psychology’ only those subfields of psychology that emulate ‘statistical thermodynamics’ in the sense that higher-level phenomena... can be explained by lower-level ... phenomena. ... I want to define psychological phenomena (percepts) of depth and motion perception, textural segmentation, focal attention, etc., as excitatory and inhibitory interactions between pools of neurons tuned to specific features (binocular disparity, motion disparity, texture gradients, etc.). This structuralist (reductionist) quest can be pursued only in vision ..., since the monkey visual system is practically identical to the human one, and is intensively studied by neurophysiologists ... We have to restrict our stimuli so that only the first retinal and cortical input stages ... are stimulated, thus we have to reduce or eliminate the influence of the higher ... cortical stages of semantic memory and symbolic processing” [13] (p. 57).

We designed Figure 3 to pay homage to two of Julesz’s main contributions, namely, (1) random-pattern stereopsis and (2) texture perception. To elaborate on item 1, we used a texture to add binocular disparity cues so as to aid the veridical depth percept and thus weaken the hollow-mask illusion [17]. To elaborate on (2), we used one of the most familiar Julesz texture pairs [18], rather than use *any* texture. The two texture regions in the pattern, separated along the vertical midline in Bela’s face, have identical second-order statistics [18], and were used by Julesz to single out the features (textons) of ‘closure’ (triangles) and ‘terminator’ (three-pronged “anchors”). This texture field is used as the carrier signal for the stereo pair that defines his facial features. In contrast to Figure 1, the texture signals in this figure provide adequate disparity cues to produce a concave percept under pseudoscopic conditions, as observed by Georgeson [17].

## Figures and Tables

**Figure 1:**
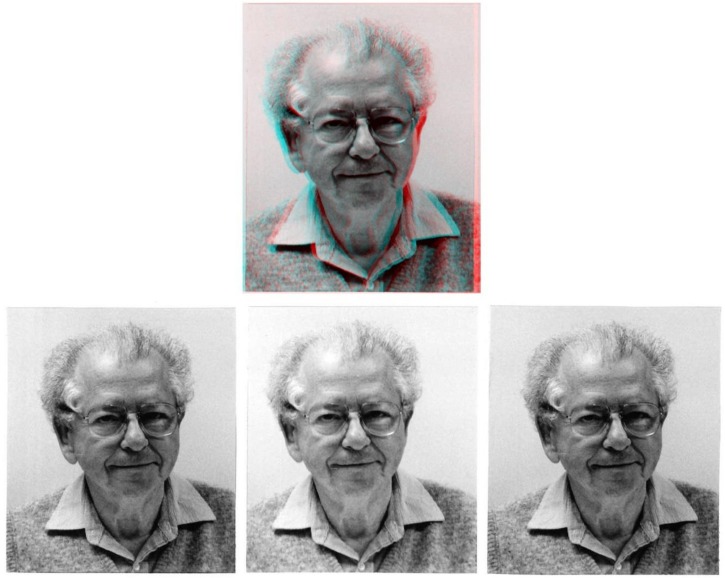
Stereoscopic photograph of Bela Julesz. The photographs were taken in the late 1990s by TVP and KM. The left and right eye views are from two locations separated by slightly more than the average interocular distance. Upper: An anaglyph in which the proper depth configuration can be seen with red/cyan glasses (red/cyan filter in front of the left/right eye, respectively). Lower: Left eye, right eye and left eye members of the stereoscopic pairs. There are two ways to fuse the two stereo sets of photographs so as to obtain the proper depth configuration, i.e., a natural face: to cross-fuse the right pair, or to uncross-fuse the left pair. A pseudoscopic pairing can be seen with the anaglyph by reversing the glasses (cyan/red filter in front of the left/right eye) or by swapping the left- and right-eye images for the bottom panel: i.e., either cross-fuse the left pair, or uncross-fuse the right pair; this has the effect of reversing the signs of the binocular disparities.

**Figure 2:**
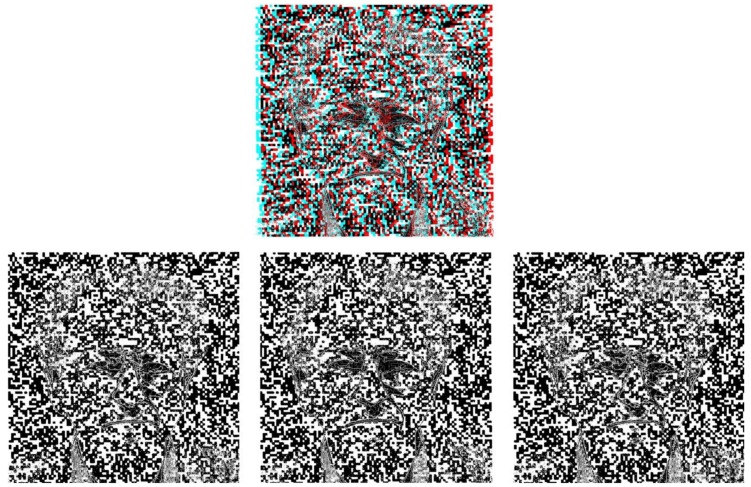
*Random-dot stereoscopist*. Stereo ‘perceptual portrait’ of Bela Julesz, based on the stereoscopic photograph shown in Figure 1, embedded in a random-dot pattern. Upper: An anaglyph which can be seen in depth with red/cyan glasses. Lower: Left-, right- and left-eye members of the stereoscopic pairs. The modes for proper and pseudoscopic depth viewing are the same as in Figure 1.

**Figure 3:**
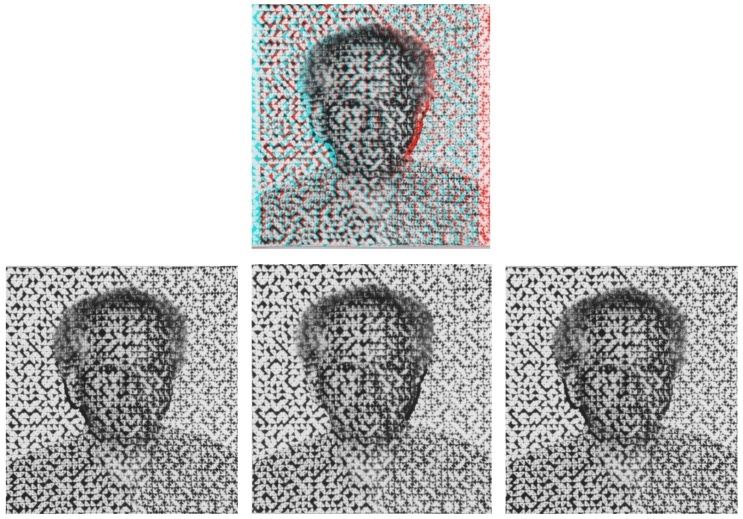
*Reductionist*. A stereo ‘perceptual portrait’ that combines elements of texture perception and random-pattern stereopsis in the Julesz style. These photographs were created by the method suggested by Georgeson [17]: We projected the texture field onto Bela’s face frontally using a slide projector while he was sitting still on a chair in a dark room, and we took two stereoscopic photographs in the same way as those in Figure 1. The conventions for the stereo pairs and the modes for proper and pseudoscopic depth viewing are the same as in Figure 1.

## References

[1] Wheatstone C. (1838). Contributions to the physiology of vision—Part the first. On some remarkable, and hitherto unobserved, phenomena of binocular vision. Philos. Trans. R. Soc..

[2] Julesz B. (1971). Foundations of Cyclopean Perception.

[3] Wade N. (1995). Psychologists in Word and Image.

[4] Brewster D. (1826). On the optical illusion of the conversion of cameos into intaglios, and of intaglios into cameos, with an account of other analogous phenomena. Edinb. J. Sci..

[5] Gregory R.L. (1970). The Intelligent Eye.

[6] Wheatstone C. (1852). Contributions to the physiology of vision—Part the second. On some remarkable, and hitherto unobserved, phenomena of binocular vision. Philos. Trans. R. Soc..

[7] Poggio G.F., Motter B.C., Squatrito S., Trotter Y. (1985). Responses of neurons in visual cortex (V1 and V2) of the alert macaque to dynamic random-dot stereograms. Vis. Res..

[8] Chubb C., Papathomas T.V., Chubb C., Gorea A., Kowler E. (1995). Motion Perception. Early Vision and Beyond.

[9] Julesz B., Payne R.A. (1968). Differences between monocular and binocular stroboscopic movement perception. Vis. Res..

[10] Braddick O. (1974). A short-range process in apparent motion. Vis. Res..

[11] Papathomas T.V., Gorea A., Julesz B. (1991). Two carriers for motion perception: Color and luminance. Vis. Res..

[12] Julesz B. (1962). Visual pattern discrimination. IRE Trans. Inf. Theory.

[13] Julesz B. (1995). Dialogues on Perception.

[14] Kovács I., Julesz B. (1994). Perceptual sensitivity maps within globally defined visual shapes. Nature.

[15] Julesz B. (1974). Cooperative phenomena in binocular depth perception. Am. Sci..

[16] Papathomas T.V., Chubb C., Gorea A., Kowler E. (1995). Early Vision and Beyond.

[17] Georgeson M.A. (1979). Random-dot stereograms of real objects: Observations on stereo faces and moulds. Perception.

[18] Caelli T., Julesz B., Gilbert E. (1978). On perceptual analyzers underlying visual texture discrimination: Part II. Biol. Cybern..

